# Crystal structure of (±)-3-[(benzo[*d*][1,3]dioxol-5-yl)meth­yl]-2-(3,4,5-tri­meth­oxy­phen­yl)-1,3-thia­zolidin-4-one

**DOI:** 10.1107/S160053681402340X

**Published:** 2014-11-05

**Authors:** Rodolfo Moreno-Fuquen, Juan C. Castillo, Rodrigo Abonia, Javier Ellena, Carlos A. De Simone

**Affiliations:** a Departamento de Química, Facultad de Ciencias Naturales y Exactas, Universidad del Valle, AA 25360, Santiago de Cali, Colombia; b Instituto de Física de São Carlos, IFSC, Universidade de São Paulo, USP, São Carlos, SP, Brazil

**Keywords:** crystal structure, benzo[*d*][1,3]dioxole, 1,3-thia­zolidin-4-one, biological properties, pharmacological properties, hydrogen bonding

## Abstract

In the title thia­zolidine-4-one derivative, C_20_H_21_NO_6_S, the central thia­zolidine ring is essentially planar (r.m.s. deviation for all non-H atoms = 0.0287 Å) and forms a dihedral angle of 88.25 (5)° with the meth­oxy-substituted benzene ring and 74.21 (4)° with the 1,3-benzodioxole ring. The heterocyclic ring (with two O atoms) fused to benzene ring adopts an envelope conformation with the non-ring-junction C atom as the flap. In the crystal, the mol­ecules are linked into chains along [001] through weak C—H⋯O inter­actions, forming *R* ^4^_4_(28) edge-fused rings.

## Related literature   

For biological and pharmacological properties of thia­zolidin-4-one systems, see: Rojas *et al.* (2011 ▶); Jackson *et al.* (2007 ▶); Gududuru *et al.* (2004 ▶); Kunzler *et al.* (2013 ▶); Rawal *et al.* (2008 ▶); Barreca *et al.* (2002 ▶); Rawal *et al.* (2007 ▶); Cunico *et al.* (2007 ▶). For similar structures, see: Fun *et al.* (2011 ▶); Cunico *et al.* (2007 ▶). For the synthesis of heterocycles of synthetic and biological inter­est, see: Abonia *et al.* (2010 ▶); Abonia (2014 ▶); Moreno-Fuquen *et al.* (2014 ▶). For hydrogen bonding, see: Nardelli (1995 ▶). For hydrogen-bond graph-set motifs, see: Etter (1990 ▶).
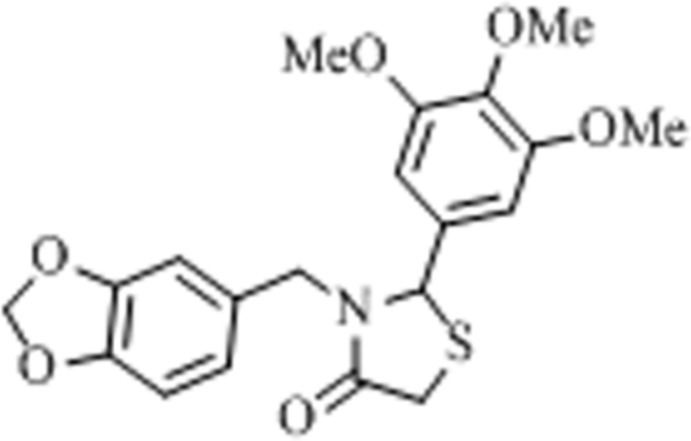


## Experimental   

### Crystal data  

C_20_H_21_NO_6_S
*M* *_r_* = 403.44
Monoclinic, 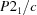

*a* = 15.3098 (11) Å
*b* = 14.3677 (12) Å
*c* = 8.6546 (3) Å
β = 97.429 (4)°
*V* = 1887.7 (2) Å^3^
*Z* = 4
Mo *K*α radiation
μ = 0.21 mm^−1^
*T* = 295 K
0.25 × 0.24 × 0.12 mm

### Data collection  

Nonius KappaCCD diffractometer
6325 measured reflections
3845 independent reflections
2922 reflections with *I* > 2σ(*I*)
*R*_int_ = 0.018

### Refinement  

*R*[*F* ^2^ > 2σ(*F* ^2^)] = 0.049
*wR*(*F* ^2^) = 0.150
*S* = 1.03
3845 reflections
258 parameters
H atoms treated by a mixture of independent and constrained refinement
Δρ_max_ = 0.34 e Å^−3^
Δρ_min_ = −0.34 e Å^−3^

### 

Data collection: *COLLECT* (Nonius, 2000 ▶); cell refinement: *SCALEPACK* (Otwinowski & Minor, 1997 ▶); data reduction: *DENZO* (Otwinowski & Minor, 1997 ▶) and *SCALEPACK*; program(s) used to solve structure: *SHELXS97* (Sheldrick, 2008 ▶); program(s) used to refine structure: *SHELXL97* (Sheldrick, 2008 ▶); molecular graphics: *ORTEP-3 for Windows* (Farrugia, 2012 ▶) and *Mercury* (Macrae *et al.*, 2006 ▶); software used to prepare material for publication: *WinGX* (Farrugia, 2012 ▶).

## Supplementary Material

Crystal structure: contains datablock(s) I, global. DOI: 10.1107/S160053681402340X/gg2142sup1.cif

Structure factors: contains datablock(s) I. DOI: 10.1107/S160053681402340X/gg2142Isup2.hkl

**Fig. 1.**
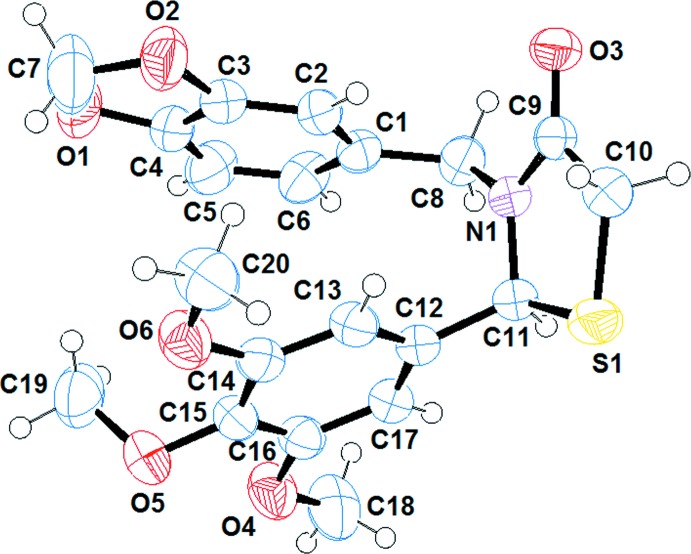
Molecular conformation and atom numbering scheme for the title compound with displacement ellipsoids drawn at the 50% probability level. H atoms are shown as spheres of arbitrary radius.

. DOI: 10.1107/S160053681402340X/gg2142fig1.tif
Mol­ecular conformation and atom numbering scheme for the title compound with displacement ellipsoids drawn at the 50% probability level. H atoms are shown as spheres of arbitrary radius.

**Fig. 2.**
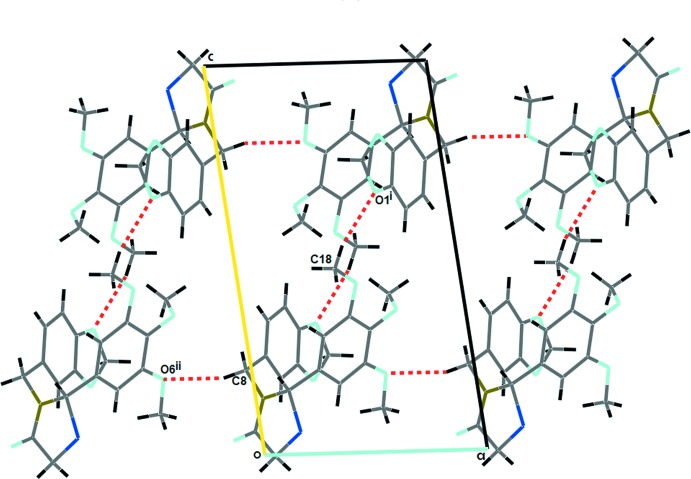
Part of the crystal structure of (I), forming one-dimensional chain, along [001]. Symmetry code: (i) -x+1,-y,-z+1; (ii) x,+y,+z-1.

. DOI: 10.1107/S160053681402340X/gg2142fig2.tif
Part of the crystal structure of (I), forming one-dimensional chain, along [001]. Symmetry code: (i) −x+1,-y,-z+1; (ii) x,+y,+z-1.

**Fig. 3.**
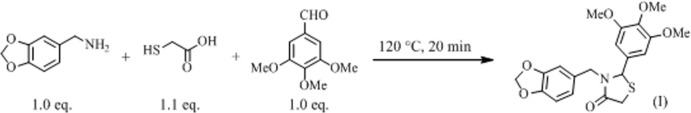
The formation of the title compound.

. DOI: 10.1107/S160053681402340X/gg2142fig3.tif
The formation of the title compound.

CCDC reference: 1030709

Additional supporting information:  crystallographic information; 3D view; checkCIF report

## Figures and Tables

**Table 1:** Hydrogen-bond geometry (, )

*D*H*A*	*D*H	H*A*	*D* *A*	*D*H*A*
C18H18*A*O1^i^	0.96	2.45	3.350(3)	155
C8H8*B*O6^ii^	0.97	2.60	3.529(2)	161

Symmetry codes: (i) 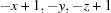; (ii) 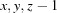.

## supplementary crystallographic information

### S1. Comment

The title thiazolidin-4-one compound, C_20_H_21_NO_6_S belongs to a class of important heterocycles that have attracted considerable attention because of their biological and pharmacological properties. Their structures are present in a well-known group of patented drugs and substances which possess antimalarial (Rojas *et al.*, 2011), anti-arrhythmic (Jackson *et al.*, 2007), antitumor (Gududuru *et al.*, 2004), antifungal (Kunzler *et al.*, 2013), antihepatitic (Rawal *et al.*, 2008) and antiviral (Barreca *et al.*, 2002) among other activities. There is an interest in developing biologically active molecules, with 5-membered rings containing two heteroatoms. Among them, the thiazolidin-4-ones are one of the most investigated classes of compounds (Rawal *et al.*, 2007). Continuing with our current studies on the use of imines and imminium ions for the synthesis of heterocycles of synthetic and biological interest (Abonia *et al.*, 2010, Abonia, 2014, Moreno-Fuquen *et al.*, 2014), the 1,3-thiazolidin-4-one (I) was obtained from a solvent-free three-component reaction involving 3,4-(methylenedioxy)benzylamine, mercaptoacetic acid and 3,4,5-trimethoxybenzaldehyde. The reaction proceeded with the initial formation of an imine, which underwent a nucleophilic attack by the sulfur atom of the mercaptoacetic acid, followed by an intramolecular cyclization with the releasing of a molecule of water to afford the title compound (I).

The molecular structure of (I) is shown in Fig. 1. The central thiazolidine (C9/C10/S1/C11/N1) ring is essentially planar [r.m.s. deviation for all non-H atoms = 0.0287 Å] and it forms dihedral angles of 88.25 (5)° with the methoxy-substituted benzene ring and 74.21 (4)° with the 1,3-benzodioxole ring. The 1,3-benzodioxole ring is essentially planar [r.m.s. deviation for all non-H atoms = 0.0439 Å]. The dihedral angle between the benzene and benzodioxole rings is 25.12 (8)°. Two methoxy groups attached to the benzene ring are approximately parallel to the plane of the ring and the third methoxy group forms a nearly perpendicular angle with this ring. Methoxy groups on the benzene ring, have the following values of torsion angles: -3.9 (3)°, 81.9 (2)° and -1.4 (3)°. Bond lengths and bond angles in the central thiazolidine ring are very close to those reported in similar structures (Fun *et al.*, 2011; Cunico *et al.*, 2007). The molecules form a one dimensional chain, through C—H···O weak interactions, (see Table 1; Nardelli, 1995). Weak C18-H18···O1 and C8-H8···O6 contacts which reinforced each other, allow the molecules to propagate, forming *R*^4^_4_(28) edge-fused rings, along [001] (Etter, 1990), (see Fig. 2).

### S2. Experimental

Reagents and solvents for the synthesis were obtained from the Aldrich Chemical Co., and were used without additional purification. A 5 mL pyrex test tube was charged with a mixture of 3,4,5-trimethoxybenzaldehyde (145 mg, 0.74 mmol), mercaptoacetic acid (75 mg, 0.82 mmol) and 3,4-(methylenedioxy)benzylamine (111 mg, 0.74 mmol) in absence of solvent. The mixture was heated in an oil bath at 120° C for 20 min until the starting materials were no longer detected by thin-layer chromatography. Then, the obtained oily material was purified by column chromatography on silica gel using a mixture of CH_2_Cl_2_/EtOAc (10:1) as eluent. White crystals of (I) suitable for single-crystal X-ray diffraction were grown by slow evaporation, at ambient temperature and in air, from a solution in ethanol [66% yield, m.p. 395 (1) K].

### S3. Refinement

All H-atoms were positioned at geometrically idealized positions [C—H = 0.93 Å for aromatic, C—H = 0.97 Å for methylene and C-H = 0.96 Å for methyl group] and refined using a riding model approximation with *U*_iso_(H) = 1.2 *U*_eq_(C), (C—H methylene and aromatic) and to 1.5 (methyl) times Ueq of the respective parent atom.

### Crystal data

**Table:**

C_20_H_21_NO_6_S	*F*(000) = 848
*M**_r_* = 403.44	*D*_x_ = 1.420 Mg m^−^^3^
Monoclinic, *P*2_1_/*c*	Melting point: 395(1) K
Hall symbol: -P 2ybc	Mo *K*α radiation, λ = 0.71073 Å
*a* = 15.3098 (11) Å	Cell parameters from 4353 reflections
*b* = 14.3677 (12) Å	θ = 2.9–26.4°
*c* = 8.6546 (3) Å	µ = 0.21 mm^−^^1^
β = 97.429 (4)°	*T* = 295 K
*V* = 1887.7 (2) Å^3^	Block, white
*Z* = 4	0.25 × 0.24 × 0.12 mm

### Data collection

**Table:**

Nonius KappaCCD diffractometer	2922 reflections with *I* > 2σ(*I*)
Radiation source: fine-focus sealed tube	*R*_int_ = 0.018
Graphite monochromator	θ_max_ = 26.4°, θ_min_ = 2.9°
CCD rotation images, thick slices scans	*h* = −19→19
6325 measured reflections	*k* = −17→14
3845 independent reflections	*l* = −10→10

### Refinement

**Table:**

Refinement on *F*^2^	Secondary atom site location: difference Fourier map
Least-squares matrix: full	Hydrogen site location: inferred from neighbouring sites
*R*[*F*^2^ > 2σ(*F*^2^)] = 0.049	H atoms treated by a mixture of independent and constrained refinement
*wR*(*F*^2^) = 0.150	*w* = 1/[σ^2^(*F*_o_^2^) + (0.0917*P*)^2^ + 0.3214*P*] where *P* = (*F*_o_^2^ + 2*F*_c_^2^)/3
*S* = 1.03	(Δ/σ)_max_ < 0.001
3845 reflections	Δρ_max_ = 0.34 e Å^−^^3^
258 parameters	Δρ_min_ = −0.34 e Å^−^^3^
0 restraints	Extinction correction: *SHELXL97* (Sheldrick, 2008), Fc^*^=kFc[1+0.001xFc^2^λ^3^/sin(2θ)]^-1/4^
Primary atom site location: structure-invariant direct methods	Extinction coefficient: 0.031 (5)

### Special details

**Table:**

Geometry. All esds (except the esd in the dihedral angle between two l.s. planes) are estimated using the full covariance matrix. The cell esds are taken into account individually in the estimation of esds in distances, angles and torsion angles; correlations between esds in cell parameters are only used when they are defined by crystal symmetry. An approximate (isotropic) treatment of cell esds is used for estimating esds involving l.s. planes.
Refinement. Refinement of *F*^2^ against ALL reflections. The weighted *R*-factor *wR* and goodness of fit *S* are based on *F*^2^, conventional *R*-factors *R* are based on *F*, with *F* set to zero for negative *F*^2^. The threshold expression of *F*^2^ > σ(*F*^2^) is used only for calculating *R*-factors(gt) *etc*. and is not relevant to the choice of reflections for refinement. *R*-factors based on *F*^2^ are statistically about twice as large as those based on *F*, and *R*- factors based on ALL data will be even larger.

### Fractional atomic coordinates and isotropic or equivalent isotropic displacement parameters (Å^2^)

**Table:**

	*x*	*y*	*z*	*U*_iso_*/*U*_eq_	
S1	0.05694 (4)	0.24960 (4)	0.17750 (7)	0.0661 (2)	
N1	0.14541 (10)	0.12826 (11)	0.03330 (17)	0.0446 (4)	
O1	0.33492 (13)	−0.23628 (12)	0.3230 (2)	0.0820 (5)	
O2	0.18594 (11)	−0.21212 (12)	0.26924 (19)	0.0699 (5)	
O3	0.03899 (10)	0.05317 (10)	−0.12885 (15)	0.0544 (4)	
O4	0.43425 (9)	0.18093 (12)	0.53026 (18)	0.0665 (4)	
O5	0.36360 (10)	0.04832 (11)	0.69166 (16)	0.0633 (4)	
O6	0.20130 (10)	−0.01846 (11)	0.59983 (16)	0.0611 (4)	
C1	0.25404 (13)	−0.00145 (13)	0.0739 (2)	0.0465 (4)	
C2	0.19505 (13)	−0.06376 (13)	0.1275 (2)	0.0471 (4)	
H2	0.1345	−0.0546	0.1076	0.056*	
C3	0.23005 (13)	−0.13912 (14)	0.2109 (2)	0.0496 (5)	
C4	0.31919 (15)	−0.15342 (14)	0.2424 (2)	0.0569 (5)	
C5	0.37858 (15)	−0.09274 (17)	0.1948 (3)	0.0681 (6)	
H5	0.4390	−0.1018	0.2186	0.082*	
C6	0.34390 (14)	−0.01630 (15)	0.1082 (3)	0.0597 (5)	
H6	0.3824	0.0262	0.0723	0.072*	
C7	0.2520 (2)	−0.2654 (2)	0.3556 (4)	0.0985 (10)	
H7A	0.2483	−0.2583	0.4660	0.118*	
H7B	0.2436	−0.3307	0.3291	0.118*	
C8	0.21892 (14)	0.08075 (14)	−0.0244 (2)	0.0517 (5)	
H8A	0.2663	0.1250	−0.0290	0.062*	
H8B	0.2001	0.0593	−0.1297	0.062*	
C9	0.06129 (12)	0.11079 (12)	−0.02699 (19)	0.0440 (4)	
C10	−0.00378 (13)	0.16964 (15)	0.0466 (2)	0.0531 (5)	
H10A	−0.0405	0.1304	0.1028	0.064*	
H10B	−0.0415	0.2033	−0.0332	0.064*	
C11	0.16404 (13)	0.19973 (13)	0.1526 (2)	0.0487 (4)	
H11	0.1991 (14)	0.2490 (14)	0.116 (2)	0.047 (5)*	
C12	0.21490 (13)	0.16198 (13)	0.3015 (2)	0.0455 (4)	
C13	0.17821 (13)	0.09318 (14)	0.3864 (2)	0.0485 (4)	
H13	0.1202	0.0744	0.3582	0.058*	
C14	0.22882 (13)	0.05301 (14)	0.5134 (2)	0.0480 (4)	
C15	0.31476 (13)	0.08397 (14)	0.5598 (2)	0.0494 (5)	
C16	0.35014 (13)	0.15420 (14)	0.4761 (2)	0.0495 (5)	
C17	0.30002 (13)	0.19268 (14)	0.3455 (2)	0.0485 (4)	
H17	0.3238	0.2388	0.2882	0.058*	
C18	0.47265 (17)	0.2509 (2)	0.4451 (4)	0.0809 (8)	
H18A	0.5313	0.2637	0.4940	0.121*	
H18B	0.4747	0.2298	0.3404	0.121*	
H18C	0.4379	0.3066	0.4432	0.121*	
C19	0.40464 (18)	−0.03832 (19)	0.6684 (3)	0.0789 (7)	
H19A	0.4372	−0.0586	0.7648	0.118*	
H19B	0.3604	−0.0837	0.6335	0.118*	
H19C	0.4440	−0.0312	0.5914	0.118*	
C20	0.11256 (16)	−0.0499 (2)	0.5620 (3)	0.0705 (6)	
H20A	0.1015	−0.0999	0.6305	0.106*	
H20B	0.0728	0.0005	0.5738	0.106*	
H20C	0.1037	−0.0715	0.4561	0.106*	

### Atomic displacement parameters (Å^2^)

**Table:**

	*U*^11^	*U*^22^	*U*^33^	*U*^12^	*U*^13^	*U*^23^
S1	0.0659 (4)	0.0607 (4)	0.0668 (4)	0.0178 (3)	−0.0108 (3)	−0.0235 (3)
N1	0.0525 (9)	0.0384 (8)	0.0411 (7)	0.0034 (7)	−0.0006 (6)	−0.0032 (6)
O1	0.0796 (12)	0.0583 (10)	0.0997 (13)	0.0094 (9)	−0.0203 (10)	0.0197 (9)
O2	0.0717 (10)	0.0571 (9)	0.0785 (10)	−0.0042 (8)	0.0007 (8)	0.0229 (8)
O3	0.0715 (9)	0.0474 (8)	0.0423 (7)	−0.0078 (7)	−0.0001 (6)	−0.0040 (6)
O4	0.0510 (8)	0.0731 (11)	0.0703 (9)	−0.0093 (7)	−0.0115 (7)	0.0114 (8)
O5	0.0688 (10)	0.0676 (10)	0.0492 (8)	0.0066 (8)	−0.0093 (7)	0.0052 (7)
O6	0.0635 (9)	0.0663 (10)	0.0527 (8)	−0.0078 (7)	0.0049 (6)	0.0109 (7)
C1	0.0514 (10)	0.0423 (10)	0.0461 (9)	0.0034 (8)	0.0072 (8)	−0.0041 (8)
C2	0.0463 (10)	0.0450 (10)	0.0487 (10)	0.0017 (8)	0.0011 (8)	−0.0004 (8)
C3	0.0566 (11)	0.0443 (10)	0.0467 (10)	−0.0019 (8)	0.0017 (8)	−0.0024 (8)
C4	0.0622 (12)	0.0446 (11)	0.0594 (12)	0.0071 (10)	−0.0093 (9)	−0.0018 (9)
C5	0.0483 (11)	0.0590 (13)	0.0941 (17)	0.0082 (10)	−0.0015 (11)	−0.0036 (12)
C6	0.0500 (11)	0.0495 (12)	0.0808 (14)	−0.0006 (9)	0.0137 (10)	−0.0004 (10)
C7	0.095 (2)	0.087 (2)	0.108 (2)	−0.0009 (17)	−0.0095 (18)	0.0476 (18)
C8	0.0570 (11)	0.0478 (11)	0.0518 (10)	0.0038 (9)	0.0122 (9)	0.0026 (8)
C9	0.0565 (11)	0.0366 (9)	0.0374 (8)	−0.0016 (8)	0.0007 (7)	0.0038 (7)
C10	0.0548 (11)	0.0517 (12)	0.0510 (10)	0.0031 (9)	0.0003 (8)	−0.0020 (9)
C11	0.0564 (11)	0.0395 (10)	0.0473 (10)	−0.0005 (8)	−0.0039 (8)	−0.0045 (8)
C12	0.0525 (10)	0.0391 (9)	0.0429 (9)	0.0016 (8)	−0.0015 (7)	−0.0054 (7)
C13	0.0488 (10)	0.0489 (11)	0.0462 (10)	−0.0018 (9)	0.0002 (8)	−0.0050 (8)
C14	0.0567 (11)	0.0456 (10)	0.0415 (9)	−0.0009 (9)	0.0060 (8)	−0.0035 (8)
C15	0.0536 (11)	0.0511 (11)	0.0414 (9)	0.0053 (9)	−0.0019 (8)	−0.0024 (8)
C16	0.0472 (10)	0.0497 (11)	0.0495 (10)	0.0007 (8)	−0.0018 (8)	−0.0062 (8)
C17	0.0524 (10)	0.0452 (10)	0.0465 (10)	0.0003 (8)	0.0009 (8)	−0.0010 (8)
C18	0.0523 (13)	0.089 (2)	0.0960 (19)	−0.0172 (12)	−0.0103 (12)	0.0222 (15)
C19	0.0738 (16)	0.0671 (16)	0.0913 (18)	0.0130 (13)	−0.0061 (13)	0.0130 (14)
C20	0.0702 (14)	0.0772 (16)	0.0645 (13)	−0.0188 (13)	0.0107 (11)	0.0061 (12)

### Geometric parameters (Å, º)

**Table:**

S1—C10	1.788 (2)	C7—H7A	0.9700
S1—C11	1.828 (2)	C7—H7B	0.9700
N1—C9	1.349 (2)	C8—H8A	0.9700
N1—C11	1.458 (2)	C8—H8B	0.9700
N1—C8	1.459 (2)	C9—C10	1.509 (3)
O1—C4	1.385 (3)	C10—H10A	0.9700
O1—C7	1.399 (4)	C10—H10B	0.9700
O2—C3	1.378 (2)	C11—C12	1.517 (3)
O2—C7	1.405 (3)	C11—H11	0.97 (2)
O3—C9	1.225 (2)	C12—C17	1.382 (3)
O4—C16	1.367 (2)	C12—C13	1.393 (3)
O4—C18	1.418 (3)	C13—C14	1.386 (3)
O5—C15	1.380 (2)	C13—H13	0.9300
O5—C19	1.420 (3)	C14—C15	1.398 (3)
O6—C14	1.369 (2)	C15—C16	1.392 (3)
O6—C20	1.429 (3)	C16—C17	1.396 (3)
C1—C6	1.386 (3)	C17—H17	0.9300
C1—C2	1.393 (3)	C18—H18A	0.9600
C1—C8	1.513 (3)	C18—H18B	0.9600
C2—C3	1.371 (3)	C18—H18C	0.9600
C2—H2	0.9300	C19—H19A	0.9600
C3—C4	1.372 (3)	C19—H19B	0.9600
C4—C5	1.361 (3)	C19—H19C	0.9600
C5—C6	1.395 (3)	C20—H20A	0.9600
C5—H5	0.9300	C20—H20B	0.9600
C6—H6	0.9300	C20—H20C	0.9600
			
C10—S1—C11	94.26 (9)	C9—C10—H10B	110.1
C9—N1—C11	119.64 (16)	S1—C10—H10B	110.1
C9—N1—C8	121.38 (16)	H10A—C10—H10B	108.4
C11—N1—C8	118.90 (16)	N1—C11—C12	112.43 (15)
C4—O1—C7	104.81 (19)	N1—C11—S1	105.27 (13)
C3—O2—C7	104.86 (19)	C12—C11—S1	114.18 (14)
C16—O4—C18	117.14 (16)	N1—C11—H11	110.3 (12)
C15—O5—C19	114.25 (18)	C12—C11—H11	107.3 (13)
C14—O6—C20	117.56 (17)	S1—C11—H11	107.2 (12)
C6—C1—C2	119.85 (18)	C17—C12—C13	120.77 (17)
C6—C1—C8	120.77 (18)	C17—C12—C11	118.81 (17)
C2—C1—C8	119.37 (17)	C13—C12—C11	120.29 (17)
C3—C2—C1	117.18 (18)	C14—C13—C12	119.46 (18)
C3—C2—H2	121.4	C14—C13—H13	120.3
C1—C2—H2	121.4	C12—C13—H13	120.3
C2—C3—C4	122.25 (19)	O6—C14—C13	124.46 (18)
C2—C3—O2	128.10 (19)	O6—C14—C15	115.25 (17)
C4—C3—O2	109.59 (18)	C13—C14—C15	120.28 (18)
C5—C4—C3	122.0 (2)	O5—C15—C16	119.59 (18)
C5—C4—O1	128.6 (2)	O5—C15—C14	120.59 (18)
C3—C4—O1	109.4 (2)	C16—C15—C14	119.76 (17)
C4—C5—C6	116.4 (2)	O4—C16—C15	115.97 (17)
C4—C5—H5	121.8	O4—C16—C17	124.14 (19)
C6—C5—H5	121.8	C15—C16—C17	119.89 (18)
C1—C6—C5	122.3 (2)	C12—C17—C16	119.78 (18)
C1—C6—H6	118.8	C12—C17—H17	120.1
C5—C6—H6	118.8	C16—C17—H17	120.1
O1—C7—O2	109.8 (2)	O4—C18—H18A	109.5
O1—C7—H7A	109.7	O4—C18—H18B	109.5
O2—C7—H7A	109.7	H18A—C18—H18B	109.5
O1—C7—H7B	109.7	O4—C18—H18C	109.5
O2—C7—H7B	109.7	H18A—C18—H18C	109.5
H7A—C7—H7B	108.2	H18B—C18—H18C	109.5
N1—C8—C1	113.99 (15)	O5—C19—H19A	109.5
N1—C8—H8A	108.8	O5—C19—H19B	109.5
C1—C8—H8A	108.8	H19A—C19—H19B	109.5
N1—C8—H8B	108.8	O5—C19—H19C	109.5
C1—C8—H8B	108.8	H19A—C19—H19C	109.5
H8A—C8—H8B	107.6	H19B—C19—H19C	109.5
O3—C9—N1	124.56 (18)	O6—C20—H20A	109.5
O3—C9—C10	122.99 (18)	O6—C20—H20B	109.5
N1—C9—C10	112.44 (15)	H20A—C20—H20B	109.5
C9—C10—S1	108.06 (14)	O6—C20—H20C	109.5
C9—C10—H10A	110.1	H20A—C20—H20C	109.5
S1—C10—H10A	110.1	H20B—C20—H20C	109.5
			
C6—C1—C2—C3	−1.2 (3)	C8—N1—C11—C12	−60.8 (2)
C8—C1—C2—C3	177.50 (16)	C9—N1—C11—S1	−2.3 (2)
C1—C2—C3—C4	0.4 (3)	C8—N1—C11—S1	174.29 (13)
C1—C2—C3—O2	−176.52 (18)	C10—S1—C11—N1	4.45 (14)
C7—O2—C3—C2	−175.1 (2)	C10—S1—C11—C12	−119.35 (15)
C7—O2—C3—C4	7.6 (3)	N1—C11—C12—C17	113.8 (2)
C2—C3—C4—C5	1.1 (3)	S1—C11—C12—C17	−126.32 (17)
O2—C3—C4—C5	178.6 (2)	N1—C11—C12—C13	−62.1 (2)
C2—C3—C4—O1	−177.80 (18)	S1—C11—C12—C13	57.8 (2)
O2—C3—C4—O1	−0.3 (2)	C17—C12—C13—C14	−2.2 (3)
C7—O1—C4—C5	174.1 (3)	C11—C12—C13—C14	173.64 (17)
C7—O1—C4—C3	−7.1 (3)	C20—O6—C14—C13	−3.9 (3)
C3—C4—C5—C6	−1.8 (3)	C20—O6—C14—C15	177.17 (19)
O1—C4—C5—C6	176.9 (2)	C12—C13—C14—O6	−176.27 (17)
C2—C1—C6—C5	0.5 (3)	C12—C13—C14—C15	2.7 (3)
C8—C1—C6—C5	−178.2 (2)	C19—O5—C15—C16	−100.9 (2)
C4—C5—C6—C1	0.9 (3)	C19—O5—C15—C14	81.9 (2)
C4—O1—C7—O2	12.0 (3)	O6—C14—C15—O5	−5.0 (3)
C3—O2—C7—O1	−12.2 (3)	C13—C14—C15—O5	175.98 (17)
C9—N1—C8—C1	−98.3 (2)	O6—C14—C15—C16	177.76 (17)
C11—N1—C8—C1	85.2 (2)	C13—C14—C15—C16	−1.3 (3)
C6—C1—C8—N1	−137.52 (19)	C18—O4—C16—C15	178.6 (2)
C2—C1—C8—N1	43.8 (2)	C18—O4—C16—C17	−1.4 (3)
C11—N1—C9—O3	178.98 (17)	O5—C15—C16—O4	2.1 (3)
C8—N1—C9—O3	2.5 (3)	C14—C15—C16—O4	179.39 (18)
C11—N1—C9—C10	−1.8 (2)	O5—C15—C16—C17	−177.93 (17)
C8—N1—C9—C10	−178.28 (16)	C14—C15—C16—C17	−0.7 (3)
O3—C9—C10—S1	−175.68 (14)	C13—C12—C17—C16	0.3 (3)
N1—C9—C10—S1	5.1 (2)	C11—C12—C17—C16	−175.61 (17)
C11—S1—C10—C9	−5.43 (15)	O4—C16—C17—C12	−178.89 (18)
C9—N1—C11—C12	122.59 (18)	C15—C16—C17—C12	1.2 (3)

### Hydrogen-bond geometry (Å, º)

**Table:**

*D*—H···*A*	*D*—H	H···*A*	*D*···*A*	*D*—H···*A*
C18—H18*A*···O1^i^	0.96	2.45	3.350 (3)	155
C8—H8*B*···O6^ii^	0.97	2.60	3.529 (2)	161

Symmetry codes: (i) −*x*+1, −*y*, −*z*+1; (ii) *x*, *y*, *z*−1.
