# Supplementary figures and images for: Crystal structure of (±)-3-[(benzo[d][1,3]dioxol-5-yl)meth­yl]-2-(3,4,5-tri­meth­oxy­phen­yl)-1,3-thia­zolidin-4-one
Source: Acta Crystallogr Sect E Struct Rep Online. 2014 Nov 5;70(Pt 12):o1235–6. doi: 10.1107/S160053681402340X (PMC4257375; doi:10.1107/S160053681402340X)

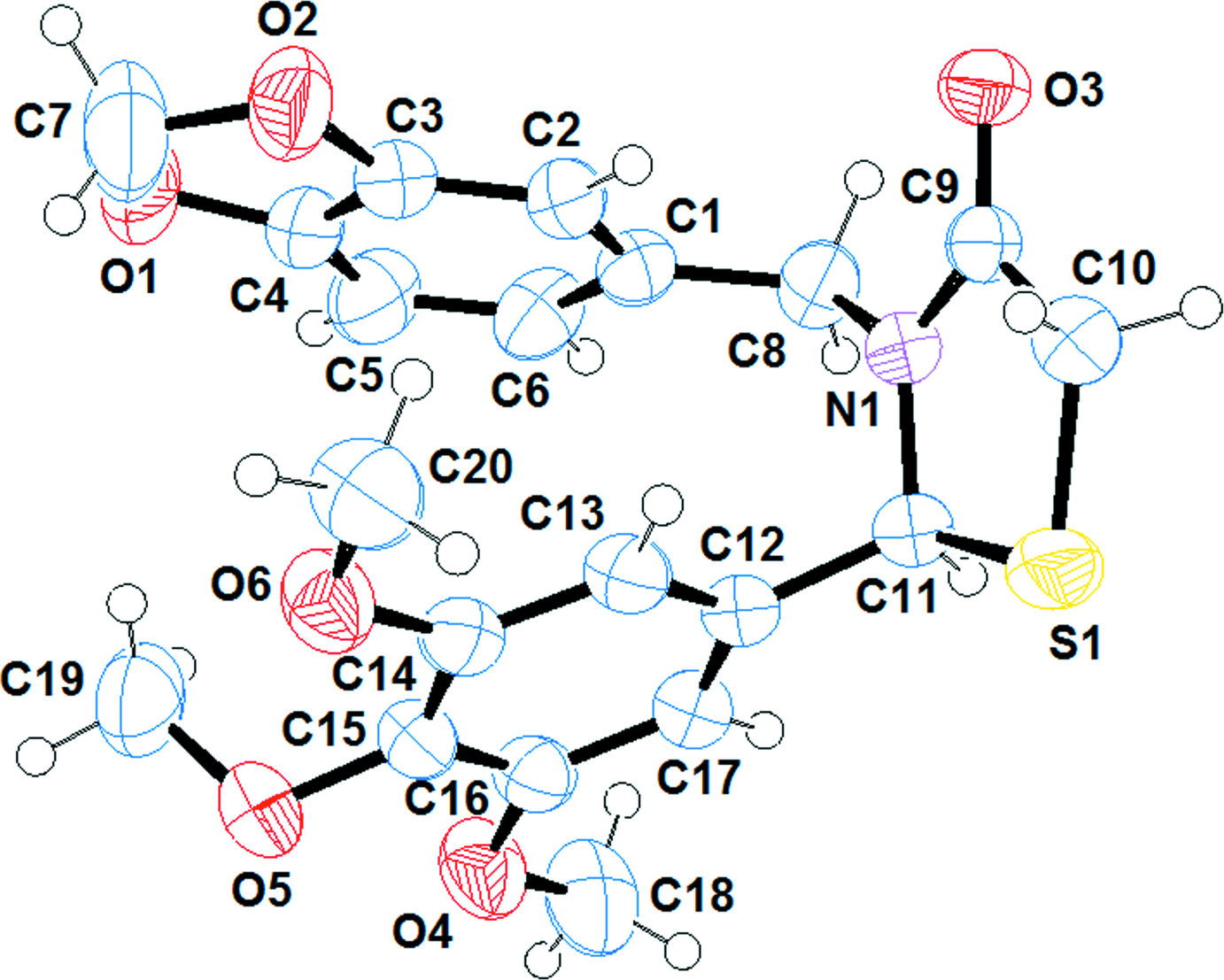

Supplement: Supplementary file 3 [file e-70-o1235-fig1.tif]

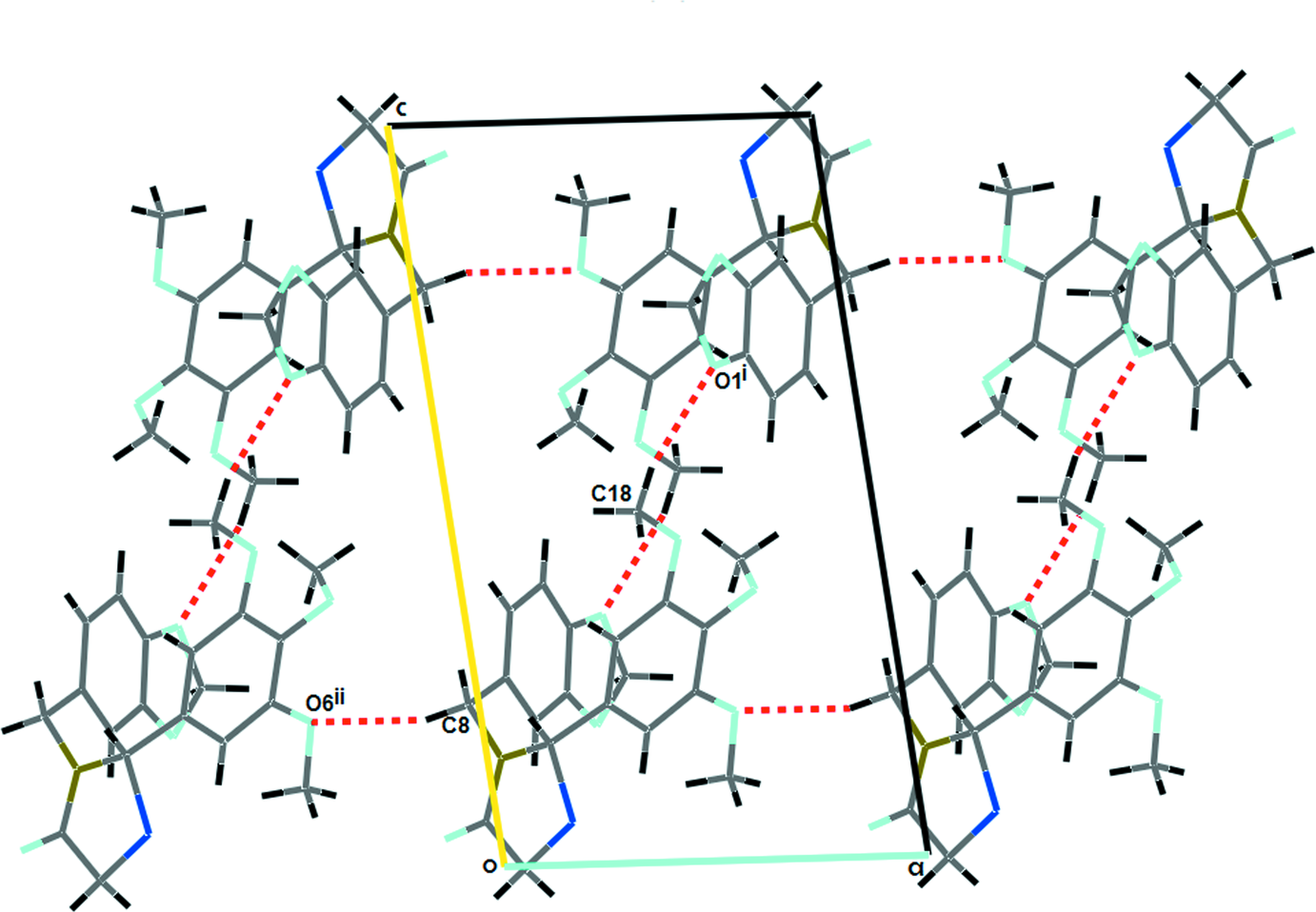

Supplement: Supplementary file 4 [file e-70-o1235-fig2.tif]

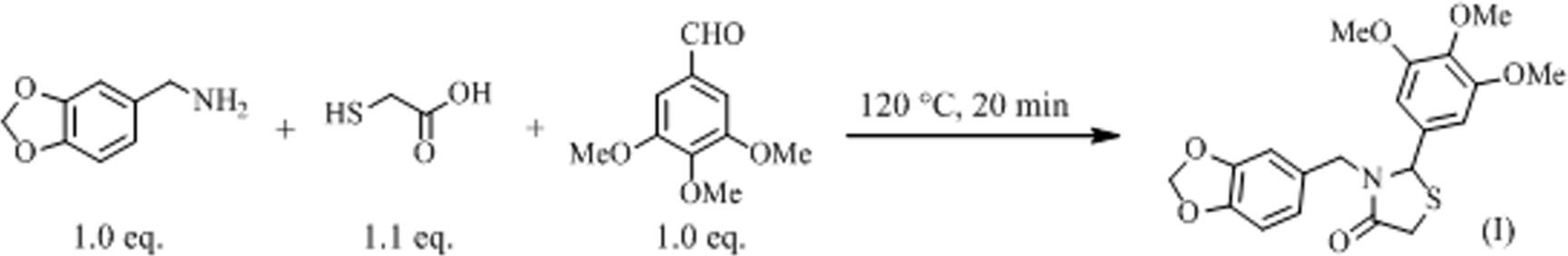

Supplement: Supplementary file 5 [file e-70-o1235-fig3.tif]
